# In situ visualization of large-scale turbulence simulations in Nek5000 with ParaView Catalyst

**DOI:** 10.1007/s11227-021-03990-3

**Published:** 2021-08-02

**Authors:** Marco Atzori, Wiebke Köpp, Steven W. D. Chien, Daniele Massaro, Fermín Mallor, Adam Peplinski, Mohamad Rezaei, Niclas Jansson, Stefano Markidis, Ricardo Vinuesa, Erwin Laure, Philipp Schlatter, Tino Weinkauf

**Affiliations:** 1 SimEx/FLOW, Engineering Mechanics, KTH Royal Institute of Technology, 100 44 Stockholm, Sweden; 2 Division of Computational Science and Technology, KTH Royal Institute of Technology, 100 44 Stockholm, Sweden; 3 PDC Center for High Performance Computing, KTH Royal Institute of Technology, 100 44 Stockholm, Sweden

**Keywords:** In situ visualization, High-performance computing, Computational fluid dynamics

## Abstract

In situ visualization on high-performance computing systems allows us to analyze simulation results that would otherwise be impossible, given the size of the simulation data sets and offline post-processing execution time. We develop an in situ adaptor for Paraview Catalyst and Nek5000, a massively parallel Fortran and C code for computational fluid dynamics. We perform a strong scalability test up to 2048 cores on KTH’s Beskow Cray XC40 supercomputer and assess in situ visualization’s impact on the Nek5000 performance. In our study case, a high-fidelity simulation of turbulent flow, we observe that in situ operations significantly limit the strong scalability of the code, reducing the relative parallel efficiency to only $\approx 21\%$ on 2048 cores (the relative efficiency of Nek5000 without in situ operations is $\approx 99\%$). Through profiling with Arm MAP, we identified a bottleneck in the image composition step (that uses the Radix-kr algorithm) where a majority of the time is spent on MPI communication. We also identified an imbalance of in situ processing time between rank 0 and all other ranks. In our case, better scaling and load-balancing in the parallel image composition would considerably improve the performance of Nek5000 with in situ capabilities. In general, the result of this study highlights the technical challenges posed by the integration of high-performance simulation codes and data-analysis libraries and their practical use in complex cases, even when efficient algorithms already exist for a certain application scenario.

## Introduction and background

The availability of high-performance computing (HPC) resources and efficient computational methods allow the study of complex turbulent flows via time-dependent high-fidelity numerical simulations. This type of flow is ubiquitous in nature as well as industrial applications, and it plays a crucial role in phenomena as diverse as atmospheric precipitations and the creation of the lift and drag forces acting on aircraft.

In the context of computational fluid dynamics (CFD), we consider both direct numerical and well-resolved large-eddy scale-resolving simulations (DNS and LES, respectively) as high-fidelity simulations, in which most of the independent degrees of freedom of the system are resolved explicitly, without the aid of modeling. In the case of turbulent flows, due to the large-scale separation in both space and time, such an approach results in computational meshes which may contain between $\approx 10^6$ and $\approx 10^9$ grid points, and simulations which proceed for $\approx 10^6$ time steps. A relevant example is the DNS of the flow around a wing profile in [11], which employed $2.3 \times 10^9$ grid points. Carrying out these studies is challenging for two reasons: on the one hand, because of computational costs of the order of multiple millions of CPU hours, and on the other hand, because the datasets created by each simulation can be as large as tens of Terabytes.

To mitigate the first difficulty, researchers have focused on developing codes with high strong scalability, which requires minimizing communication and load imbalance between nodes, as discussed, e.g., by Merzari et al. [16] and Offermans [18]. This approach results in software packages that, although they often employ sophisticated numerical strategies, are relatively simple and can be used efficiently on a large number of cores. In particular, CFD codes are often limited to the solution of partial differential equations and do not provide data analysis or visualization tools. Nek5000 [8], which we consider in this work, is one of such codes. Because the software employed for the actual simulation is not equipped with tools for post-processing, the typical workflow followed by CFD researchers requires to externally store intermediate datasets, which are the input for further analysis. This standard procedure does not have a significant drawback when the intermediate datasets are small, as in cases when only time-independent statistics are retained. However, the possibility of carrying out more complex post-processing analysis, such as to study the time evolution of topological features, is limited by the second obstacle mentioned above, i.e., very high input/output (I/O) requirements. The in situ methodology, which consists of coupling a simulation code with a set of libraries for data analysis, is a natural strategy to overcome this difficulty, but it will become a viable option for CFD researchers only if the efficiency and scalability of their code are preserved.

In situ methods for flow simulations have gradually matured over the years since the potential of coupling visualization with simulations was first demonstrated in the 1990s (Haimes [10]; Ma [14]). With extreme-scale on the horizon, Ma [15] presented the challenges and opportunities of in situ visualization, later realized by Rasquin et al. [23], combining both in situ visualization with computational steering, running the flow solver PHASTA on 160k cores, connected to ParaView running on a separate visualization cluster. More recently, using Catalyst, Yi et al. [29] demonstrated that both simulations, visualization, and steering could be executed on the same computational resources. The feasibility of extreme-scale in situ processing was later demonstrated by Ayachit et al. [2], running PHASTA using SENSEI and Catalyst for in situ visualization on more than 1 million MPI ranks, achieving a low 13% in situ overhead. Specifically regarding Nek5000, Damaris/Viz [6] performed in situ visualization using VisIt, and compared time-partitioning and space-partitioning and the visualization operation is a volume slice. Color plots were also used by Bernardoni et al. [4] who present a new adaptor for Nek5000 using SENSEI. Furthermore, Nek5000+SENSEI and ParaView/Catalyst were used for mesh validation by Shudler et al. [25], who also performed a scalability test up to 420 processes, but without a direct comparison of the same simulation with and without in situ. At a similar time as Bernardoni et al. [4] and Shudler et al. [25], we started to work on a new in situ adaptor for Nek5000 and a standard version of Paraview/Catalyst, which does not require the use of SENSEI for data transfer. In this paper, we present our implementation, and we describe in detail the impact of a reasonably complex in situ visualization on the computational cost of the simulation. Note that the test case that we employ is closer to a full-scale high-fidelity numerical simulation than those in Refs. [4, 25]. Furthermore, the in situ operations that we perform include a three-dimensional visualization at higher resolution and the computation of a scalar quantity in the entire domain, which makes our data analysis more computationally intensive. The three main contributions of this work are:

1. We design and implement in situ visualization with Paraview Catalyst [1, 3] in Nek5000 [9], a widely-used and Gordon-Bell award winner Fortran/C CFD code. To achieve this, we design and implement a C++ Catalyst adaptor in Nek5000 and a visualization and data analysis pipeline in Python. The test case that we examined consists of a CFD simulation of realistic size; alongside ParaView is employed for a standard visualization of vortex clusters in turbulent flows.
2. We measure and analyze the parallel performance of Nek5000 with in situ operations when running up to 2048 cores on a Cray XC40 supercomputer, identifying the aggregation step in the visualization pipeline as the major obstacle to achieve strong scalability.
3. We use the Arm MAP profiler to identify precisely the in situ and message-passing interface (MPI) functions that are causing performance degradation. We find that the parallel implementation of the Radix-kr algorithm (used for image composition) [17, 22] in Paraview Catalyst is responsible for time spent in MPI communication.

We summarized how the most recent related works differ from the present one in Table 1.

**Table 1:** Overview of prior work on in situ visualization use cases and integrations for massively parallel computational fluid dynamics codes

CFD code	In situ coupling	Analysis/vis	Hardware	Number of cores
PHASTA [29]	ParaView/Catalyst	Vorticity, Slice	Titan (Cray XK7), Mira (IBM BlueGene/Q)	up to 32,768
PHASTA [2]	SENSEI with ParaView/Catalyst	Slice	Mira (IBM BlueGene/Q)	up to 1,048,576
Nek5000 [6]	Damaris/Viz with VisIt	Slice	stremi/Grid’5000 (HP ProLiant)	up to 816
Nek5000 [4]	SENSEI with VisIt/LibSim	Histogram, Slice	*not specified*	*not specified*
Nek5000 [25]	SENSEI with ParaView/Catalyst	Clipping	Cooley (Intel Haswell)	up to 420
Nek5000 (**Ours**)	ParaView/Catalyst	Magnitude, Isosurface	Beskow (Cray XC40, Intel Haswell)	up to 2048

The paper is organized as follows. Section 2 provides an overview of Nek5000 and explains the different steps in designing and implementing in situ visualization in Nek5000. Section 3 presents the experimental set-up we carry out our performance measurements. In Sect. 4, we describe the performance and scaling results together with information from a parallel profiler. Finally, Sect. 5 summarizes the paper and draws conclusions.

## Methodology

While in situ visualization promises a significant reduction of I/O and improves overall execution performance (simulation and post-processing), co-processing itself inevitably introduces overhead to code execution. In other words, an excessive overhead during the in situ analysis and visualization step, despite its benefit, can hurt the performance and scalability of the simulation. To understand the impact of in situ visualization on parallel scientific applications, we use a CFD code called Nek5000 and implement an in situ visualization adaptor using Paraview Catalyst. To evaluate the impact on execution performance, we run a strong scaling test to understand simulation performance with and without in situ visualization. Hereafter, we provide a brief description of the two software we consider, of our in situ implementation and resulting workflow.

### Considered software

The development of Nek5000 started in the late 1980s [9] and is still in progress today [18, 19, 21, 26]. The code consists of approximately 100,000 lines of code and is written mainly in Fortran77 (70,000 lines of code) and C (30,000 lines of code). To achieve massive parallelism, the code uses MPI for parallel communication. The Nek5000 algorithm is based on the so-called spectral-element method [20], a high-order variant of the finite-element method. Accordingly, the governing equations are solved in weak form, and the discretization is implemented following the Galerkin method [5]. In practice, the computational domain is divided into quadrilateral (2D simulations) or hexahedral elements (3D simulations) and, within the elements, the solution is represented by Lagrangian interpolants. In the present project, we employed the $P_N-P_{N-2}$ formulation, i.e. in each element velocity and pressure are defined along each of the three directions on *N* points with Gauss–Lobatto–Legendre distribution and $N-2$ points with Gauss–Legendre distribution, respectively. For all the cases, we selected $N=12$, meaning that the velocity is represented with polynomials of the 11th order. Together with the accuracy and low numerical dissipation characteristic of high-order methods, Nek5000 exhibits remarkable scaling capability. For instance, El Khoury et al. [7] observed linear scaling from 8192 to 65,536 cores on DNS of the turbulent flow across a circular pipe employing more than $2 \times 10^9$ grid points.

We enable in situ visualization in Nek5000 using ParaView Catalyst. ParaView (and the included in situ library Catalyst) [1] is an open-source data analysis and visualization tool geared towards large scientific data sets based on the Visualization Toolkit (VTK) [24]. It is written in C++ but also provides bindings for other languages to facilitate large-scale software development. With a custom adapter in place to translate relevant simulation data into VTK data structures, Catalyst steers an in-place analysis and visualization through a pipeline. Traditional visualization is typically a post-processing step that is decoupled to the main simulation. In other words, the development of a visualization pipeline is often decoupled with the simulation workload. ParaView Catalyst enables this flexibility by decoupling the Catalyst Adaptor and the actual implementation of the pipeline. Instead of including the pipeline as part of the simulation and adaptor code, they are written in a separated Python script using the ParaView Python interface. The script defines the steps in the visualization pipeline and is executed by the Catalyst adaptor during co-processing. In this work, we used the ParaView GUI client to interactively generate a pipeline script using a sample dataset (data from one time step of a simulation).

### In situ implementation

One challenge of using ParaView Catalyst for in situ visualization for large-scale simulation is to have all the relevant components readily compiled and linked. In the case of Nek5000, one additional challenge is to integrate the Fortran based simulation code with a Catalyst Adaptor written in C++. However, this can be readily achieved through an additional wrapper that encapsulates and exposes Catalyst adaptor functions to the simulation code, where VTK data structures are constructed and registered for co-processing. Thereafter, a visualization pipeline can be separately constructed in a Python script (that will be used by the adaptor) using the ParaView Python interface to define the visualization workflow.

**Fig. 1:**
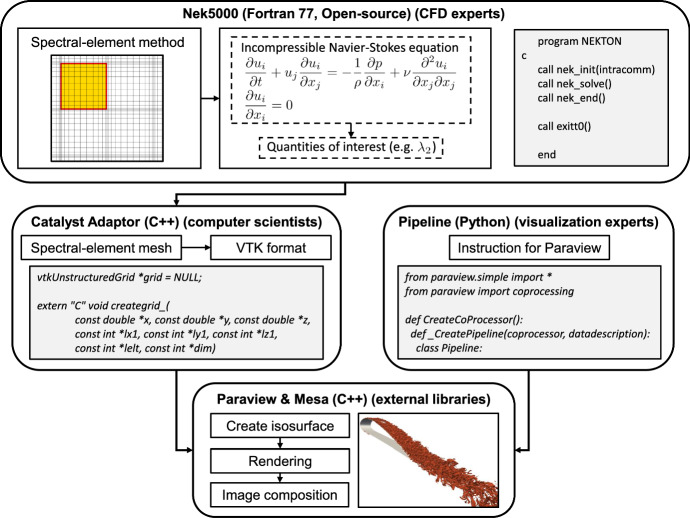
Workflow of in situ visualization of an *IsoLambda2* simulation using a Catalyst adaptor and a pipeline script. The visualization pipeline describes the configuration and steps (such as how data is processed and rendered) using the ParaView Python interface

We describe the workflow of in situ visualization in our code with Fig. 1. A simulation is initiated in Nek5000 with all the relevant initial conditions provided, the simulation initializes and proceeds to time stepping. After each time step has been computed, the simulation code calls the DoCoprocessing() function through the Fortran adaptor and provides the Catalyst Adaptor with data structures (in VTK) that are necessary for the visualization. The Nek5000 grid is a collection of structured sub-grids, each corresponding to one spectral element, with duplicated points at the elements’ boundaries. The VTK grid is created mapping the spectral-element grid in an unstructured grid, which is assembled organizing each element sub-grid in quadrilaterals (2D simulations) or hexahedrons (3D simulations). When called for the first time, the Catalyst Adaptor reads a user-provided Python script to initialize a co-processor. Our script defines, among other settings, at which time step interval the visualizations are created, how the output image is rendered (camera position, image size, transfer function, etc.), and how the data is processed (e.g., which iso value is used). The data structures provided by the simulation code to the Adaptor are processed through the pipeline and eventually writes an output image to disk. After the initial invocation, the Catalyst Adaptor only needs to update the co-processor using the latest data for relevant time steps and the pipeline will be invoked. One exciting feature of in situ visualization with Catalyst is the possibility to stream data directly to a ParaView GUI client for live visualization during a simulation. However, our focus is on writing visualization to files in this work.

**Fig. 2:**
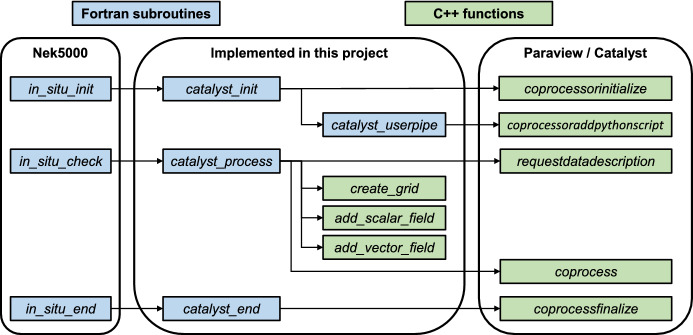
Structure of the in situ adaptor implemented in this project. A more detailed description is provided in the repository documentation at https://github.com/KTH-Nek5000/InSituPackage

In our implementation, we took advantage of the fact that general placeholder subroutines for in situ operations are already present in Nek5000. In particular, these Fortran subroutines include (1) in situ initialization (*in_situ_init*), which is performed once, after a preliminary time step and before the beginning of the actual time loop; (2) the in situ processor (*in_situ_check*), which is performed at the end of each time step; and (3) insitu finalization (*in_situ_end*), which is performed once, after the end of the time loop. We implemented three corresponding Fortran subroutines, which call standard VTK functions and the additional ones developed during the project (written in C++). The function *catalyst_init* corresponds to *in_situ_init*, and it includes the initialization of the Paraview coprocressor and the reading of the visualization pipeline. The function *catalyst_process* corresponds to *in_situ_check*, and it includes most of the operation. In this function, a VTK grid is created, organizing the spectral-element mesh in Nek5000, and the set of required scalars and vector fields are mapped into the VTK grid (e.g., pressure, velocity, and $\lambda_2$). Furthermore, the Paraview coprocessor is called, which executes the instructions in the visualization pipeline. Lastly, the function *catalyst_end* corresponds to *in_situ_end*, and it includes the finalization of the in situ coprocessor. The structure of the in situ adaptor implemented in this project is illustrated in Fig. 2.

## Experimental setup

**Fig. 3:**
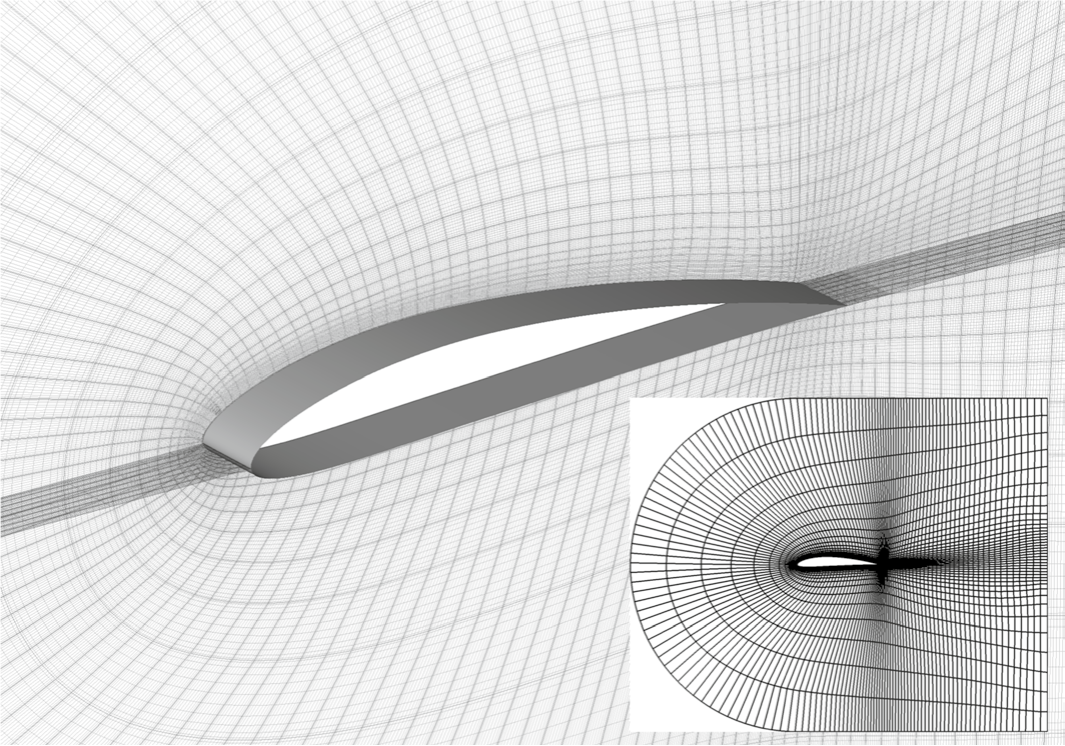
Detail of the mesh in the proximity of the NACA4412 airfoil and (insert) side view of the computational domain. Note that in the side view only the spectral elements are shown

We present a test case that is designed to be of the size as a small but still realistic numerical simulation carried out in a typical research project, and much larger than a tutorial case. The simulation is a highly resolved LES, which describes the incompressible flow around a NACA4412 airfoil at a chord Reynolds number of $Re_c=100{,}000$ ($Re_c=U_\infty c/ \nu$, where $U_\infty$ is the incoming velocity of the flow at a large distance from the airfoil, *c* is the airfoil chord length, and $\nu$ is the fluid kinematic viscosity). The computational domain extends in any direction for at least 2*c* from the airfoil (see Fig. 3), and appropriate boundary conditions (BCs) are imposed [28], to have a consistent velocity distribution. The resolution to accurately simulate the turbulent flow requires a total of $48\times 10^6$ grid points. Note that this case was included in a study of the flow around a NACA4412 airfoil up to $Re_c=1{,}000{,}000$ [28], and that the simulations at higher $Re_c ,$ and thus higher resolutions were designed following the same methodology. We refer to Refs. [27, 28] for a more detailed description of the setup, relevance, and physical results.

**Fig. 4:**
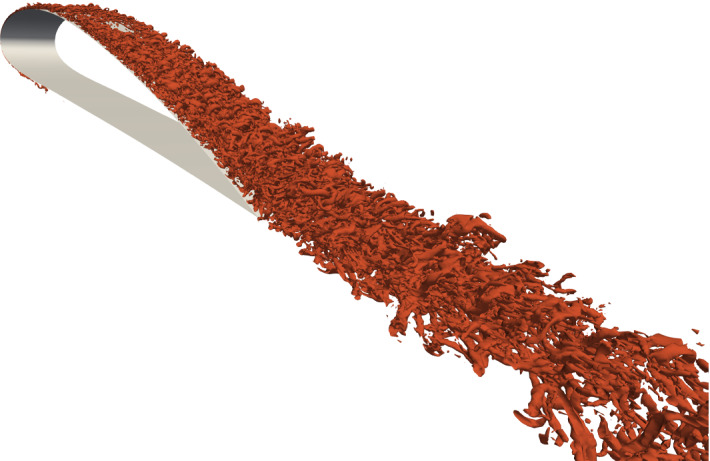
Iso-surface of the $\varvec{\lambda }_{\mathbf {2}}$ criterion [12] to identify near-wall vortical structures for the 3D turbulent flow around a NACA 4412 wing section

We consider a pipeline that computes the iso-surface of the $\lambda_2$ criterion [12] at a single threshold $\lambda _2=-200U^2_\infty /c^2$ that highlights vortical structures in the wing boundary layer. Additionally, we use an iso-surface of velocity magnitude close to 0 to extract the wing surface for additional context. Note that our adapter makes the flow pressure available as well, but the presented pipeline does not use this additional field.

The iso-surface computation involves the extraction of geometric primitives i.e. triangles and quadrilaterals and can be done locally on each processor without communication with processors holding adjacent data points. The visualization of iso-surfaces in a distributed setting thus entails that each processor computes and renders the geometry for its subset of the data. Then the partial representations are combined into an image compositing step and saved to disk. We render images at a *Full HD+* resolution of $1920\times 1280$ pixels. An exemplary output image is shown in Fig. 4.

It is important to note that our choice of the task performed via Catalyst is the result of a compromise between having a simple and relatively general study case and using the in situ implementation in a meaningful way. The capability of avoiding an intermediate dataset on disk is particularly significant if the post-processing requires data with high temporal frequency, which is not the case of producing a few static figures. Nevertheless, our experiment still allows comparing the increase in computational cost using an in situ implementation, with the storage required to perform the same operation with traditional post-processing, which is our aim.

We perform all the simulations on the Beskow supercomputer at the PDC Centre for High-Performance Computing (PDC-HPC) at the KTH Royal Institute of Technology. Beskow is a Cray XC40 system, based on Intel Xeon E5-2698v3 16-core (2.30 GHz) processors and Cray Aries interconnect network with Dragonfly topology. Each Beskow node has 32 cores divided between two sockets, with 16 cores on each. The RAM for each node is 64 GB. The total number of cores is 53,632. We do not use hyperthreading when conducting the experiments. We build ParaView 5.6.3 with default parameters, together with the graphic library Mesa 18.3.3 using the Intel compiler 19.0.1.144, the build-process manager CMake 3.15.3, and Python 3.6.5.7. Nek5000 is also built with the Intel compiler 19.0.1.144. The full build process is described in “Appendix”.

## Results

We carry out a strong scalability test for the pipeline described in Sect. 3, performing a single simulation with $n_{\mathrm{steps}}=1000$ for $n_{\mathrm{CPU}}=256$, 512, 1024 and 2048 cores.

The Catalyst visualization pipeline is executed once every 50 time steps. We do not perform any additional I/O to avoid interfering with the benchmark.

**Fig. 5:**
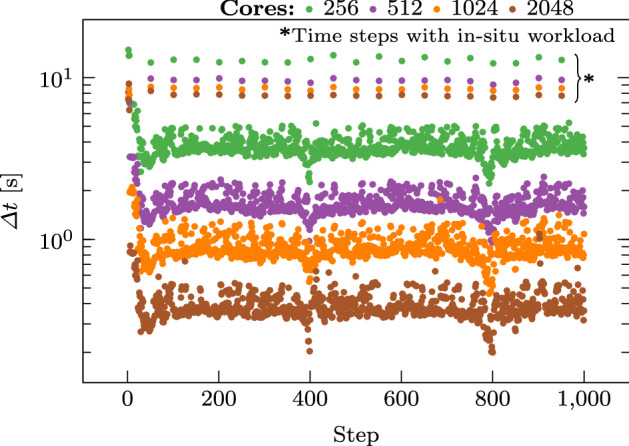
Average execution time per process on each time step over the entire simulation for $\mathbf {n=1000}$ in log scale. Different numbers of scaling configurations, 256 (green), 512 (purple), 1024 (orange), and 2048 (brown) cores, are used in each test case (color figure online)

We show in Fig. 5 the average execution time per core during each time step, denoted by $\left\langle \varDelta t \right\rangle$, for different numbers of cores. Qualitatively, the expected inverse relation between execution time and the number of cores for strong scalability holds: An increase in the number of processors leads to a decrease in execution time. Time steps when visualizations are created and saved to disk (as annotated with Time steps with in situ workloads) are immediately apparent, showing a clear spike in execution time.

**Fig. 6:**
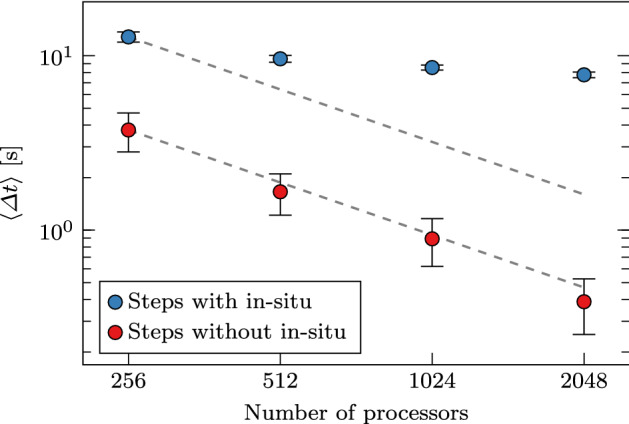
Scaling of the mean wall-time per process when scaling the number of processes. The time steps with and without in situ operations are marked in blue and red respectively. A log scale is used and error bars indicate the 95% confidence interval

Similar to Fig. 5, we report the average execution time per process during each time step with and without in situ operations, denoted by $\left\langle \varDelta t \right\rangle$, in Fig. 6. For time steps without in situ processing, $\left\langle \varDelta t \right\rangle$ decreases from $\approx 3.7$ s to $\approx 0.47$ s when the number of cores increases from 256 to 2, 048, with a relative parallel efficiency of $\approx 99\%$. However, for time steps with in situ processing, $\left\langle \varDelta t \right\rangle$ only decreases from $\approx 13$s to $\approx 7.7$s, with a relative parallel efficiency of $21\%$. At the same time, the in situ approach has an overhead of between $\approx 4.8\%$ and $\approx 31\%$ when using 256 and 2048 cores, respectively. We define overhead as the difference between the average execution time overall processes, with and without in situ processing and normalized by the latter. The total overhead (in terms of extra computation time) over the entire simulation depends on how frequently in situ processing is performed. Given that a single flow field in double precision has a size of $\approx 2.9$ GB, the in situ approach results in a reduction of $\approx 2.9 \times 1000/50=58$ GB of required storage space. Furthermore, we observe that altering the frequency of in situ processing yields negligible changes of $\left\langle \varDelta t \right\rangle$ (not shown here). With the same test case, we can estimate that using in situ analysis once every two time steps (which would save 1459 GB of storage) will result in an overhead of $\approx 120\%$ and $\approx 780\%$ when using 256 and 2048 cores, respectively. The increasing overhead per increasing number of cores is the consequence of coupling codes with different scalability properties.

**Fig. 7:**
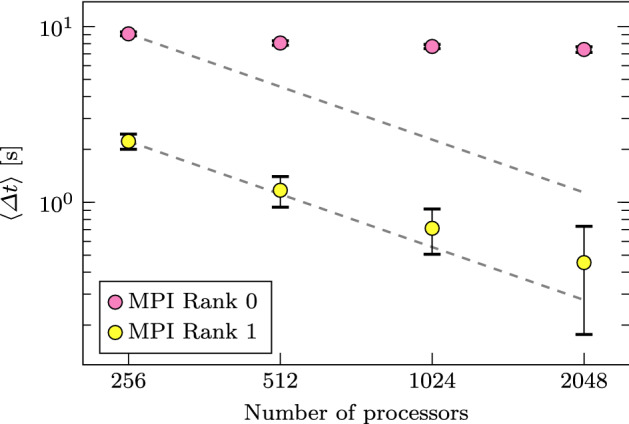
Execution time per time step for the in situ visualization pipeline, for MPI rank 0 (pink) and MPI rank 1 (yellow). Results from other ranks are not reported as they behave similarly to rank 1. A log scale is used and error bars indicate the 95% confidence interval.

To investigate the lack of scalability and attempt to identify the bottleneck in Catalyst/ParaView, we measure the time spent in pipeline execution for different MPI ranks. We observe a remarkable imbalance between rank 0 and all other rank and report the differences in Fig. 7. Interestingly, not only the time spent by the pipeline in ranks different than 0 is lower, but it also shows a better scaling. Our results show that rank 0 is a major bottleneck in the in situ processing pipeline. We initially suspected image writing to the file system to be the cause of this. However, we used ParaView with the default setting for image composition and verified that the bottleneck is not I/O related. For this reason, we suspected the image composition itself to have caused the bottleneck. In particular, the performance scaling of all other ranks than rank 0 suggests that the compute workload is well distributed, indicating the collection (assembly) to be an issue, i.e., a part of the in situ implementation is apparently working as a serial code.

**Fig. 8:**
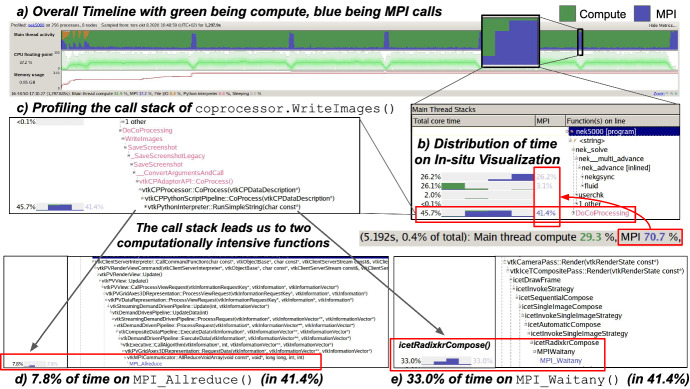
**a** Profiling of a full Nek5000 simulation using Arm MAP allows us to distinguish between compute and MPI workloads. **b** The time distribution reveals that MPI accounts for approximately 70.7% of the execution time when co-processing is active (where 41.4% is from co-processing). **c** We investigate the source of the bottleneck by expanding the call stack of the in situ processing function and find that **d** a MPI_Allreduce is taking 7.8% of the time. **e** However, a MPI_Waitany that is used in the image composition inside the ICE-T library (icetRadixkrCompose) accounts for 33.0% of the time

To further explore our observation with regard to the load imbalance between rank 0 and all other ranks, we profile a full simulation using the Arm MAP profiler^1^ on Beskow using 256 processes on eight compute nodes. MAP is a low-overhead profiler that enables performance analyses of compute and MPI activities in HPC applications. We present an extract of the profiling results in Fig. 8. The execution timeline in Fig. 8a shows five peaks where MPI activity is dominant, representing both the in situ visualization (DoCoprocessing) and synchronization (nekgsync) step. We zoom into the timeline of interest where in situ visualization is active in Fig. 8b and notice that at the selected frames, MAP reports $70.7\%$ of the execution time is spent on MPI. This can be explained by the activity breakdown, of which 41.4% (out of 70.7%) of the MPI activities come from the visualization pipeline. At the same time, we can visually confirm that MPI communication (with blue color in the timeline) is dominating the visualization step, indicating a potential bottleneck in the pipeline. To investigate the sources of the bottleneck, we expand the call stack of the in situ processing function, DoCoprocessing, in Fig. 8c. The total core time breakdown there reveals that the WriteImages operation in the Python co-processing pipeline is solely responsible for the $41.4\%$ time spent on communication. We continue to expand the stack to locate the source of the bottleneck and eventually arrive at two MPI calls that can explain over 40% of the MPI time. Firstly, we observe that MPI_Allreduce (7.8%) (Fig. 8d) is used by VTK to perform the reduction in the update data step; secondly, we notice that a large portion of time is spent on MPI_Waitany (33.0%) (Fig. 8e) that is used in the image composition step (icetRadixkrCompose) [17, 22]. In conclusion, the MPI_Waitany is mainly responsible for the bottleneck and it indicates a major bottleneck in the image composition algorithm.

## Discussion and conclusions

The rationale for adopting the in situ approach is to avoid saving an intermediate dataset for post-processing, which may lead to considerable I/O requirements. However, in situ operations inevitably have an impact on the overall computational cost. The goal of our current effort is to investigate how these two contradictory constraints balance for a realistic high-fidelity numerical simulation. We implemented an adapter for the CFD code Nek5000 that organizes the data in VTK format, thus making it possible to use ParaView as an in situ post-processing tool through the Catalyst API. The test case that we employed is a highly-resolved LES of the turbulent flow around a wing profile, using approximately $48\times 10^6$ grid points. This is the size of a small but still realistic numerical simulation carried out in turbulence research [28].

Nek5000 exhibits approximately linear scaling when no in situ analysis is performed, however, when the in situ analysis is performed, we observed that the time per time step becomes significantly higher and it scales poorly when the number of cores increases. At 2048 cores, we only observe a relative parallel efficiency of $\approx 21\%$. For these reasons, the usage of the in situ approach is practical only in two extreme cases: (1) for a relatively large simulation and very low frequency of operations, i.e. when the overhead is negligible, and the storage of even a few fields is not possible; and (2) for a relatively small simulation, if a high frequency of operation is needed, and there is a severe storage limitation, i.e., when the much higher but yet reasonable computational cost is preferable than saving a large dataset.

To understand the lack of strong scalability, we perform detailed timing and profiling. Timing of co-processing on individual processes reveals that part of the in situ pipeline is executed by a single MPI process with rank 0, spending considerably more time than other processes. This suggests that part of the pipeline is serialized, thus limiting the achievable parallel speedup (Amdahl’s law). To pinpoint the issue, we used Arm MAP to perform profiling and discovers that a majority of the co-processing time is spent on MPI communication. Further investigation shows that the time is spent on an MPI_Waitany in the image composition step (called icetRadixkrCompose). Radix-k (and its variant Radix-kr) is an advanced algorithm for large-scale image composition. Being a computation and communication-intensive workload, the algorithm has been subjected to numerous optimization efforts [13, 17, 22]. In particular, the algorithm enables a tunable parameter *k* to adapt to the system’s interconnect topology. For example, previous works [13] have performed auto-tuning on the *k* value for higher performance, but its impact reported in Ref. [13] is almost negligible compared with the overhead of in-situ operations in our case. In this work, we have used the default parameters provided by ParaView. Likely, an improved parallel algorithm in Catalyst for the aggregation step, e.g. non-blocking or a highly distributed image composition and auto-tuning of multiple parameters, would lead to a considerable parallel performance gain.

Despite our observations, it is possible that modifications to the pipeline code or even better-optimized settings could improve the performance significantly. If this is the case, it is important to recognize that simplification of the building process of data-analysis software is itself a goal worth pursuing. At the time of writing, we have been in contact and communicated our findings with the ParaView Catalyst developers to further scrutinize the results, and more work will be needed in the future. A more general consideration is that the availability of test cases of practice relevance, e.g., medium-size numerical simulations for CFD researches, is important to help the adoption of any data-analysis methodology in new areas, as well as to identify directions of possible performance improvement. Such improvements will likely ease the effort required and facilitate the uptake in adopting these new data analysis methods in the research community.

## Appendix: Sample workflow

A sample setup of our workflow is available at: https://github.com/KTH-Nek5000/InSituPackage. The repository contains the used versions of Mesa, ParaView, and Nek5000 along with our additions and some sample pipelines. A simpler version of the test case used here is provided as well. A reduction in $Re_c$ to 75,000 compared to 100,000 used in the presented experiments and in the resolution requirements allow for the example in the repository to be run on a regular work station. Instructions to set up all dependencies and run the test case are included.

## Footnotes

1 https://www.arm.com/products/development-tools/server-and-hpc/forge/map.
